# Thermodynamic and Kinetic Analysis of Galactose Oxidase Direct Electron Transfer on Carboxyl-Terminated SAM-Modified Gold Electrodes

**DOI:** 10.3390/molecules31040694

**Published:** 2026-02-17

**Authors:** Martha Leticia Jiménez-González, Gilberto Rocha-Ortiz, Luis Gabriel Talavera-Contreras, Jose de Jésus Gómez-Guzmán, René Antaño-Lopez, Marisela Cruz-Ramírez, Luis Ortiz-Frade

**Affiliations:** 1 Departamento de Electroquímica, Centro de Investigación y Desarrollo Tecnológico en Electroquímica S.C. Parque Tecnológico Querétaro, Sanfandila, Pedro de Escobedo 76703, Querétaro, Mexico; 2 Departamento de Química, Universidad Autónoma de Aguascalientes, Av. Universidad 940, Aguascalientes 20100, Ags, Mexico; 3 Departamento Académico de Biotecnológicas y Ambientales, Universidad Autónoma de Guadalajara, Av. Patria 1201, Zapopan 45129, Jalisco, Mexico; 4 Colegio de Bachilleres, Universidad Autónoma de Querétaro, Campus San Juan del Río, Calle Corregidora No. 4, Colonia Centro, San Juan del Río 76800, Querétaro, Mexico

**Keywords:** galactose oxidase, thiocarboxylic acids, modified gold electrode, self-assembled monolayers, thermodynamic analysis

## Abstract

This study addresses the thermodynamic aspects of galactose oxidase (GAOx) adsorption and redox behavior on gold electrodes modified with self-assembled monolayers (SAMs) derived from thiocarboxylic acids, namely N-acetyl-L-cysteine (NAC), mercaptosuccinic acid (MSA), mercaptoacetic acid (MAA), and L-cysteine (Cys). The electrochemical response of GAOx immobilized on these SAM-modified surfaces was analyzed to extract key thermodynamic parameters governing enzyme–electrode interactions, including the formal redox potential (E°), surface excess (Γ), potential of zero charge (E_zc_), adsorption free energy (∆G_add_), differential capacitance (C_dl_), and surface tension (γ). The results demonstrate that the nature of the terminal functional group of the SAM significantly influences the thermodynamic stabilization of GAOx at the gold interface. Shifts in the redox potential are attributed to specific coordination and electrostatic interactions between the SAM functional groups and the GAOx metal center, leading to distinct interfacial energy landscapes. Overall, the SAM-modified electrodes provide a well-defined thermodynamic framework to probe enzyme orientation, interfacial charge distribution, and stabilization of the redox-active state of GAOx during direct electron transfer. These results offer guidelines based on thermodynamic and kinetic principles for customizing enzyme–electrode interfaces, which can enhance the efficiency, stability, and consistency of third-generation electrochemical biosensors.

## 1. Introduction

D-Galactose is a crucial aldohexose involved in cellular metabolism and is an important component of the human diet, primarily derived from lactose and, to a lesser extent, from various fruits and vegetables [1]. In addition to its nutritional value, D-galactose acts as a biologically active signaling molecule that influences metabolic pathways and helps mediate responses to stress [2]. However, high levels of galactose can lead to chronic health issues such as galactosemia [3], galactose intolerance, and citrin deficiency [4]. Moreover, elevated concentrations of D-galactose have been associated with accelerated brain aging due to the production of reactive oxygen species (ROS), which may result in cellular damage [5]. Consequently, it is vital to establish reliable techniques for accurately measuring galactose in biological samples to aid in the prevention and diagnosis of these health conditions.

To detect galactose, two main types of methodologies are employed. The first category includes techniques such as HPLC, spectrophotometry, or gravimetry; although effective, these approaches tend to be costly and often necessitate sample pretreatment [6,7,8]. The second category comprises enzymatic methods where Galactose oxidase (GAOx) is combined with a transducer to create electrochemical biosensors that have shown to be rapid and reliable for quantifying galactose levels [6,9].

First-generation electrochemical biosensors either measure cosubstrate consumption or product formation. For example, one approach involves monitoring oxygen consumption with a Clark-type oxygen electrode that correlates with galactose concentration or detecting hydrogen peroxide generation using a platinum-polarized electrode [10,11,12]. Second-generation biosensors utilize various low molecular weight molecules as redox mediators attached to the electrode surface. These mediators facilitate electron transfer between the active site of the enzyme and the electrode, allowing redox reactions at lower overpotentials [9,12,13,14,15]. Third-generation biosensors function through direct electron transfer (DET) between the enzyme’s redox-active site and the electrode, offering advantages like enhanced selectivity and simplified designs by reducing component complexity; however, documentation on them remains limited [6].

The DET process involving metalloenzymes has gained significant attention due to its importance in third-generation bioelectrochemical sensors and systems. Recent advancements in protein engineering and interfacial design reveal that alterations at the metal coordination site and protein scaffold can influence electron transfer rates by modifying redox potentials and transport pathways [16]. In copper-containing enzymes like galactose oxidase, these effects are particularly sensitive to local electrostatic conditions and interfacial arrangements [17]. Despite recent developments in artificial metalloenzymes and modeling protein-electrode interfaces, achieving efficient DET continues to present challenges due to factors such as protein shell insulation, unfavorable adsorption geometries, highly reactive prosthetic groups’ presence, and heterogeneous populations of enzymes on electrode surfaces [18]. These challenges underscore the necessity for integrated thermodynamic and kinetic analyses when designing GAOx/SAM interfaces for DET-based biosensing applications [19,20].

In addressing these challenges, self-assembled monolayers (SAMs) on electrode surfaces have emerged as a common strategy in developing third-generation electrochemical biosensors [21]. SAMs provide an effective means for enzyme immobilization while ensuring consistent layers that allow some control over enzyme distribution and orientation [19]. Specifically tailored carboxyl-terminated SAMs can modify GAOx’s interfacial microenvironment, affecting both adsorption thermodynamic stability and DET efficiency.

Despite demonstrating DET capabilities with GAOx more than twenty years ago, research into using SAMs for GAOx immobilization within third-generation biosensors has not been extensively explored [19]. Some studies have suggested modifications like employing TiO_2_ nanorod array-modified FTO electrodes [22] or glassy carbon electrodes enhanced with graphene composite films containing gold nanoparticles for this purpose [23]. Nevertheless, among various molecules used for forming SAMs on gold electrodes, thiols stand out because they effectively facilitate direct electron transfer due to strong interactions formed between sulfur atoms in thiol groups and gold atoms via chemisorption, resulting in stable Au-S bonds (40–50 kcal/mol). Such interactions promote highly ordered molecular layers, especially when multidentate thiols are utilized [23,24,25,26].

While fundamental electrochemical parameters such as standard potential (E°) and heterogeneous electron transfer rate constant (k_s_) have been reported regarding GAOx immobilization on thiol-modified electrodes, comprehensive thermodynamic characterization connected with direct electron transfer related specifically to carboxyl-terminated self-assembled monolayer chemistry remains insufficiently documented [27,28,29,30,31,32]. This study aims to investigate the electrochemical behavior of several gold electrodes modified with SAMs made from N-Acetyl-L-cysteine (NAC), mercaptosuccinic acid (MSA), mercaptoacetic acid (MAA), along with L-cysteine (Cys), onto which GAOx was immobilized—a configuration not previously recorded in the literature. The results concerning these modified electrodes will include findings about redox potential (E°), electron transfer coefficient (α), heterogeneous electron transfer rate constant (k_s_), surface excess (Γ), differential capacitance (C_dl_), zero-charge potential (E_zc_), adsorption energy (∆G_add_), along with surface tension (γ). The data derived from these parameters will provide essential insights into coating film uniformity alongside metalloenzyme orientation at the electrode surface concerning redox potential during DET processes.

## 2. Results and Discussion

### 2.1. Modification of Gold Electrodes

Cyclic voltammetry and electrochemical impedance spectroscopy were conducted on gold electrodes using a 5 mM solution of Fe(CN)_6_^3−/4−^ in a 0.1 M phosphate buffer (pH 7.2). This investigation focuses on the creation of self-assembled monolayers utilizing mercaptoacetic acid (designated as Au-MAA), L-cysteine (Au-Cys), N-acetyl-L-cysteine (Au-NAC), and mercaptosuccinic acid (Au-MSA) as surface modifiers.

**Figure 1:**
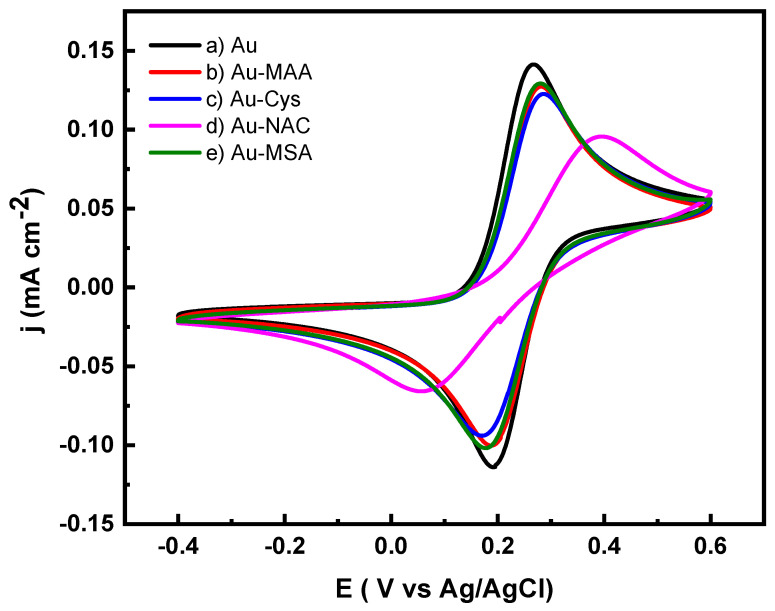
Cyclic voltammetric responses of (a) Au, (b) Au-MAA, (c) Au-Cys, (d) Au-NAC, and (e) Au-MSA electrodes in 5 mM of Fe(CN)_6_^3−/4−^ at a scan rate of 50 mV·s^−1^ using 0.1 M phosphate buffer (pH 7.2), as the supporting electrolyte.

The cyclic voltammogram for the unmodified Au electrode (Line a of Figure 1) displayed a reversible redox behavior with a ΔE_p_ = 66 mV. In comparison, the modified gold electrodes (lines b–e of Figure 1) demonstrated reduced current responses along with a transition toward quasi-reversible electron transfer characteristics [31]. Notably, the Au-NAC electrode (line d of Figure 1) showed the most pronounced reduction in current value and exhibited the largest ∆E_p_. Literature suggests that electron transfer at these modified gold electrodes, when interacting with this redox probe, may occur through tunneling across the self-assembled monolayer, diffusion of electroactive species through the monolayer, or movement through pinholes present on the electrode surface [33].

**Figure 2:**
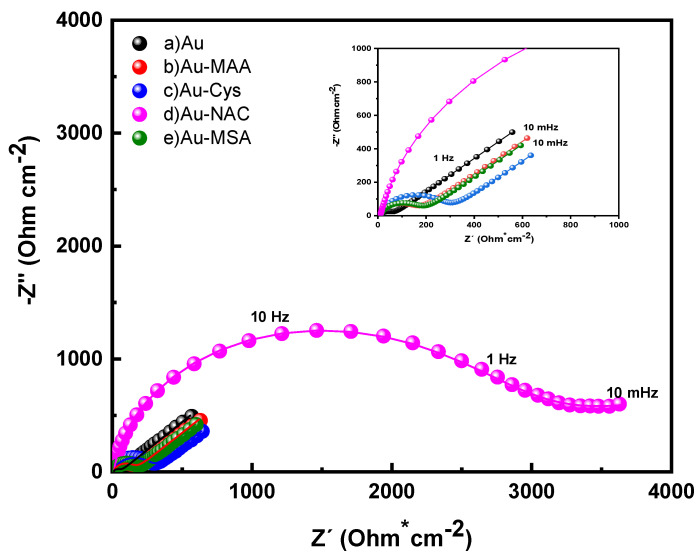
Impedance spectra of (a) Au, (b) Au-MAA, (c) Au-Cys, (d) Au-NAC, and (e) Au-MSA electrodes in 5 mM of Fe(CN)_6_^3−/4−^ solution at open circuit potential, measured over a frequency range of 10 kHz to 10 mHz with an amplitude of 10 mV.

Nyquist diagrams were generated for electrodes made of Au, Au-MAA, Au-Cys, and Au-NAC in the presence of 5 mM Fe(CN)_6_^3−/4−^ within 0.1 M phosphate buffer solutions at pH 7.2, as illustrated in Figure 2. The impedance spectra reveal a semicircular shape in the high-frequency region, indicative of the charge-transfer mechanism, followed by a straight line with a 45° angle at lower frequencies that is characteristic of the Warburg element, representing semi-infinite diffusion of electroactive species [31].

For the bare Au electrode (line a of Figure 2), charge-transfer resistance was noted across the frequency range from 10 kHz to 100 Hz, with a semicircle diameter measuring 10 Ω·cm^−2^. This observation suggests rapid electron-transfer kinetics for the Fe(CN)_6_^3−/4−^ redox couple [34]. After modifying the Au electrode with thiol self-assembled monolayers (SAMs) (lines b–e of Figure 2), the frequency range associated with charge transfer expanded compared to that of the bare Au electrode. This finding confirms successful SAM formation on the gold surface and its influence on electron-transfer dynamics. Among all the modified electrodes, although similar changes in charge-transfer behavior were observed for each thiol layer, the Au-NAC (line d of Figure 2) displayed a charge-transfer resistance that was an order of magnitude greater than that of the other electrodes, indicating a distinctly impeded electron-transfer process.

The acquired spectra were analyzed using the Randles equivalent circuit model to ascertain the proportion of the electrode surface that was coated, based on charge-transfer resistance values (Equation (1)) [34,35]. The coated fractions for Au-MAA and Au-MSA were found to be comparable, measuring at 65.9 ± 7.5% and 67.4 ± 5.3%, respectively. In contrast, the Au-Cys electrode demonstrated a significantly higher coated fraction of 81.72 ± 6.3%. This increased surface coverage observed in Au-Cys, when compared to Au-MAA and Au-MSA, is likely due to electrostatic interactions between COO^−^ and NH_3_^+^ groups, which facilitate the development of a denser film.

(1) $$\theta_{Is}^{R}=1-\frac{R_{ct}^{Au}}{R_{ct}^{SAM}}$$

Among the various electrodes modified with SAM, Au-NAC demonstrated the highest coating percentage at 97.5 ± 4.8%. This suggests that the thiol produced a more uniform and defect-free film compared to others. The observed behavior can be explained by the longer molecular chain length of NAC, which enhances the electrostatic interactions among adjacent molecules.

### 2.2. Galactose Oxidase Electron Transfer Process

**Figure 3:**
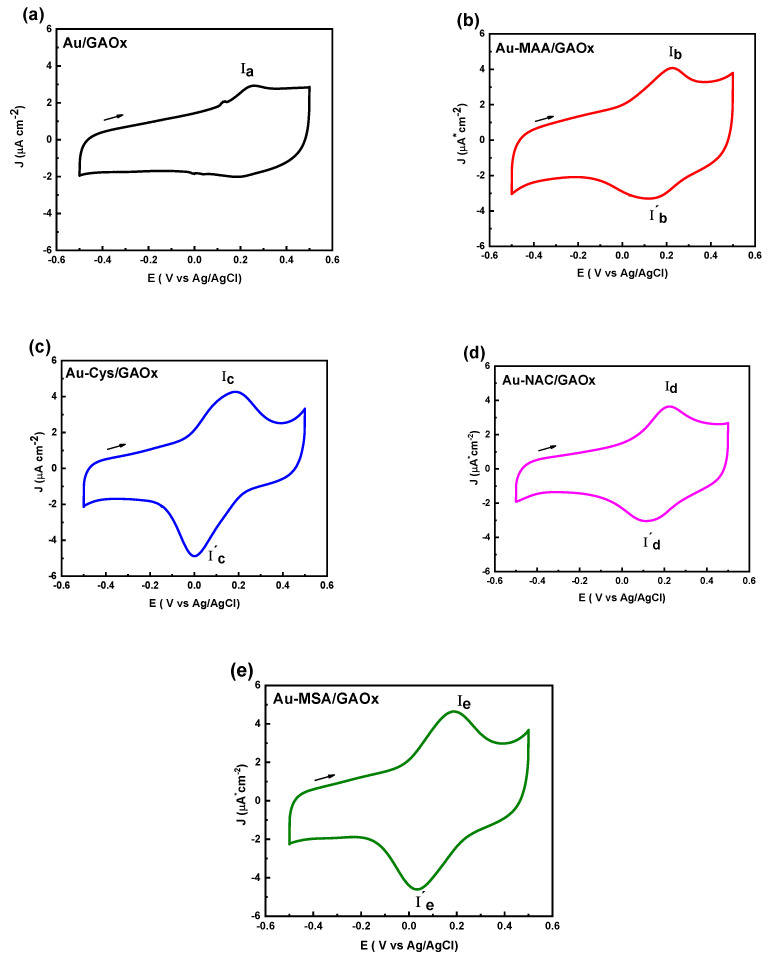
Cyclic voltammetric responses of (**a**) Au/GAOx; (**b**) Au-MAA/GAOx; (**c**) Au-Cys/GAOx; (**d**) Au-NAC/GAOx; and (**e**) Au-MSA/GAOx electrodes at a scan rate of 100 mV·s^−1^, using 0.1 M phosphate buffer (pH 7.2) as supporting electrolyte.

Cyclic voltammetry was conducted over a potential range of −0.5 to 0.5 V vs. Ag/AgCl to assess the capacity of MAA, Cys, NAC, and MSA films in promoting electron transfer within the GAOx modified electrode (Figure 3). In the case of the Au/GAOx electrode (Figure 3a), the lack of a reduction peak indicated an irreversible electron transfer process. Conversely, an oxidation peak (E_pa_) was noted at 0.2 V vs. Ag/AgCl, which is attributed to the oxidation of the GAOx active site.

The Au-MAA/GAOx electrode (Figure 3b) demonstrated a greater current response compared to the bare gold electrode, presenting an oxidation peak (E_pa_) at 0.23 V vs. Ag/AgCl. Additionally, a reduction peak (E_pc_) was observed at 0.12 V vs. Ag/AgCl, reflecting the redox activity associated with the GAOx active center. Similarly, enhancements in electron transfer were seen with Au-Cys/GAOx (Figure 3c), Au-NAC/GAOx (Figure 3d), and Au-MSA/GAOx (Figure 3e) electrodes when compared to Au/GAOx electrodes; these variations displayed both oxidation and reduction signals. The oxidation peaks were identified at 180 mV for Au-Cys, 230 mV for Au-NAC, and 190 mV for Au-MSA vs. Ag/AgCl, while their corresponding reduction peaks appeared at 2 mV, 115 mV, and 35 mV vs. Ag/AgCl respectively.

The redox potential for Cu(II)-Tyr/Cu(I)-Tyr concerning GAOx is documented as being at 0.067 V vs. Ag/AgCl (KCl saturated), while the Cu(II)-Tyr•/Cu(II)-Tyr process (Equation (2)) occurs at a potential of 0.241 V vs. Ag/AgCl (KCl saturated), as referenced in existing literature [26,36]. From these observations, we suggest that electron transfer within this study’s system arises from the redox activity of the metal center in the enzyme via the following reaction: Cu(II)-Tyr + e^−^ → Cu(I)-Tyr. Moreover, coordination between carboxyl groups and the metal center results in alterations to the redox potential values.

(2) $$\;Cu(II)-\mathrm{Tyr}\;•\overset{\pm e-(0.241V)}{↔}\;Cu(II)-\mathrm{Tyr}\overset{\pm e-(0.067V)}{↔}Cu(I)-\mathrm{Tyr}$$

**Figure 4:**
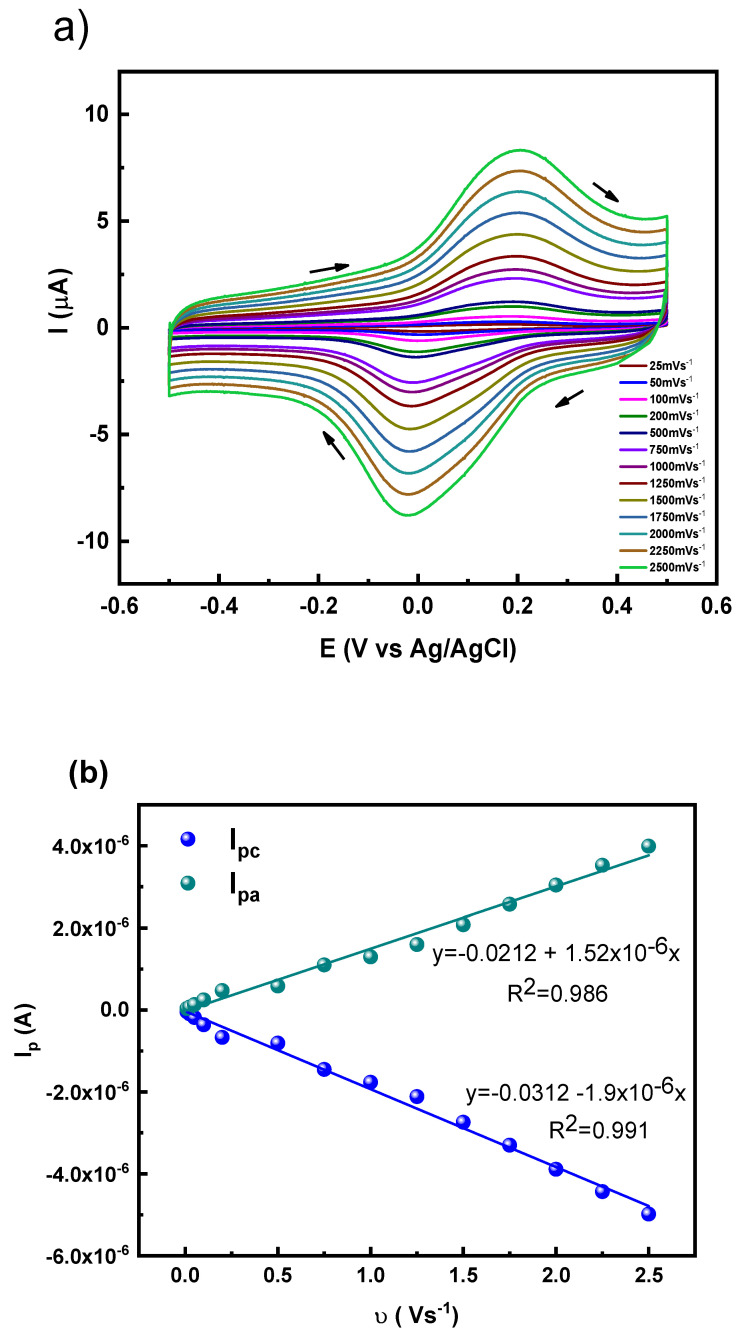
(**a**) Cyclic voltammetric response of the Au-Cys/GAOx electrode in the presence of 50 μM GAOx at different scan rates (25 to 2500 mV·s^−1^); (**b**) plot of peak currents vs. scan rate derived from the cyclic voltammograms.

Cyclic voltammetry experiments were conducted at scan rates ranging from 25 to 2500 mV·s^−1^ to evaluate the electron transfer characteristics of GAOx. The modified electrodes were pre-equilibrated in a 50 μM GAOx solution prepared in phosphate buffer (0.1 M, pH 7.2) at open-circuit potential (OCP) for 15 min to allow adsorption and stabilization of a surface-confined redox population. Subsequently, the electrodes were gently rinsed with protein-free buffer and transferred to fresh supporting electrolyte without GAOx for the scan-rate-dependent cyclic voltammetry measurements. The voltammograms for the gold electrode modified with a cysteine self-assembled monolayer (SAM) in the presence of GAOx (Figure 4a) displayed a consistent increase in both oxidation and reduction peak currents as the scan rate (v) increased, which confirms successful enzyme immobilization on the electrode surface. A similar trend was observed for electrodes modified with Au-MAA/GAOx (Figure S1a in Supplementary Materials), Au-NAC/GAOx (Figure S2a), and Au-MSA/GAOx (Figure S3a). Laviron’s model [37] was utilized to analyze I_p_ versus *v* plots for each of these electrodes—Au-Cys/GAOx (Figure 4b), AU-MAA/GAOx (Figure S1b), AU-NAC/GAOx (Figure S2b), and Au-MSA/GAOx (Figure S3b), to determine important parameters such as the electron transfer coefficient (α), surface excess concentration (Γ), and heterogeneous electron transfer rate constant (k_s_). The findings regarding these parameters for the modified electrodes, in contrast to the irreversible electrochemical response recorded for the bare gold electrode, indicate that thiol modifications significantly enhance electron transfer between the electrode and the enzyme. Additionally, a comparable electrochemical response was observed when transferring these electrodes into a pure electrolyte solution, suggesting a strong affinity of the enzyme for the modified electrode surface. A summary of results, including E° and ∆E_p_ values for Au/GAOx, Au-MAA/GAOx, Au-Cys/GAOx, Au-NAC/GAOx, and Au-MSA/GAOx electrodes can be found in Table 1.

According to earlier studies, various factors affect the values of ∆E_p_ and E°, such as the distance from the electrode surface to the enzymatic active center, the interactions between the active site and promoters, as well as the enzyme’s orientation [21,38,39]. The active center of GAOx features a square-pyramidal configuration, which comprises a copper ion coordinated with two tyrosine residues, two histidine residues, and a water molecule [40]. It has been suggested that the terminal carboxyl (COO^−^) groups of promoters engage with the copper ion at the GAOx active site by replacing the water molecule that is coordinated [19]. This hypothesis aligns with the redox potential results from this study, where MAA showed the highest value, while Cys had the lowest. This observed trend can be linked to varying inductive effects that influence electron density across the oxygen atoms in COO^−^ groups of promoters, corresponding to their pK_a_ values (pK_a_ = 3.7 for MAA and pK_a_ = 1.8 for Cys). The similarity in $k_{s}$ values suggest comparable electron transfer efficiencies among the different thiol-modified electrodes, which may indicate similar effective electronic coupling between the electrode surface and the GAOx active site. However, it is acknowledged that $k_{s}$ reflects not only distance effects but also protein orientation, orientation heterogeneity, and electronic rearrangement [41,42].

Finally, to investigate the denaturation of GAOX upon adsorption, additional electrochemical stress tests were conducted. Figure S4 illustrates the Au-Cys/GAOx electrode undergoing chronoamperometric polarization at progressively negative and then positive potentials in comparison to those used during the thermodynamic assessment. Following this chronoamperometric polarization, a slight reduction in current density was noted, along with a minimal shift in the formal potential and a partial change in the capacitive region. These alterations are attributed to interfacial reorganization and partial reorientation of the adsorbed GAOx molecules, which can influence the electronic coupling between the copper active site and the electrode surface. Nevertheless, the overall voltammetric characteristics and reversibility remain intact, indicating that the redox center is preserved and that no significant denaturation occurs under the applied polarization conditions. This observation implies that the enzyme maintains its electroactive and functional structure. Resembling behaviors seen in other thiol-modified electrodes.

### 2.3. Thermodynamic Study of Galactose Oxidase Immobilization

To achieve a more thorough understanding of the chemical species involved in the adsorption process during various stages of molecular assembly, a thermodynamic analysis was conducted on GAOx immobilization on Au electrodes using different promoters. This analysis utilized differential capacitance measurements at a frequency of 50 Hz in the presence of GAOx in solution (50 μM) [41]. These measurements are instrumental in providing insights into the orientation of chemical species present at the electrode surface, allowing for the calculation of important parameters such as charge density, surface tension, surface excess, and adsorption free energy. Understanding these parameters enhances comprehension of the interaction mechanisms between promoters and GAOx, thereby offering a clearer picture of the immobilization process. This method has seen extensive use in antigen–antibody sensors and in the immobilization of various inorganic and organic compounds [39,42,43,44,45,46,47,48,49,50,51,52,53,54].

Differential capacitance (C_dl_) measurements were taken within a potential range that corresponds to the double-layer charge region for thiols attached to the electrode surface without GAOx. Figure S5 illustrates representative C_dl_ vs. E (Ag/AgCl) plots for bare Au (Figure S5a), Au modified with MAA (Figure S5b), Au modified with cysteine (Figure S5c), Au modified with NAC (Figure S5d), and Au modified with MSA (Figure S5e) electrodes immersed in 0.1M PBS at pH 7. For unmodified Au electrodes, C_dl_ values varied between 8.92 and 9.34 μF·cm^2^, peaking at −260 mV vs. Ag/AgCl, which aligns with crystallographic reconstruction observed on the Au (111) surface [46,48,49]. Conversely, when modified with thiol coatings, a notable decrease in C_dl_ was recorded: values ranged from 9.38 to 5.38 μF·cm^2^ for Au/MAA, from 7.20 to 5.58 μF·cm^2^ for Au/Cys, from 5.45 to 3.90 μF·cm^2^ for Au/NAC, and from 7.55 to 6.10 μF·cm^2^ for Au/MSA. This decline can be attributed to the establishment of a thiol monolayer on the gold electrode that behaves like a capacitor. According to Equation (3), capacitance is influenced by both the dielectric constant (ε) and thickness (d) of thiol films [42]. An increase in thickness due to the thiol layer presence results in diminished capacitance, consistent with expectations regarding insulating films on electrode surfaces.

(3) $$C=\frac{\varepsilon \varepsilon_{0}A}{d}$$

**Figure 5:**
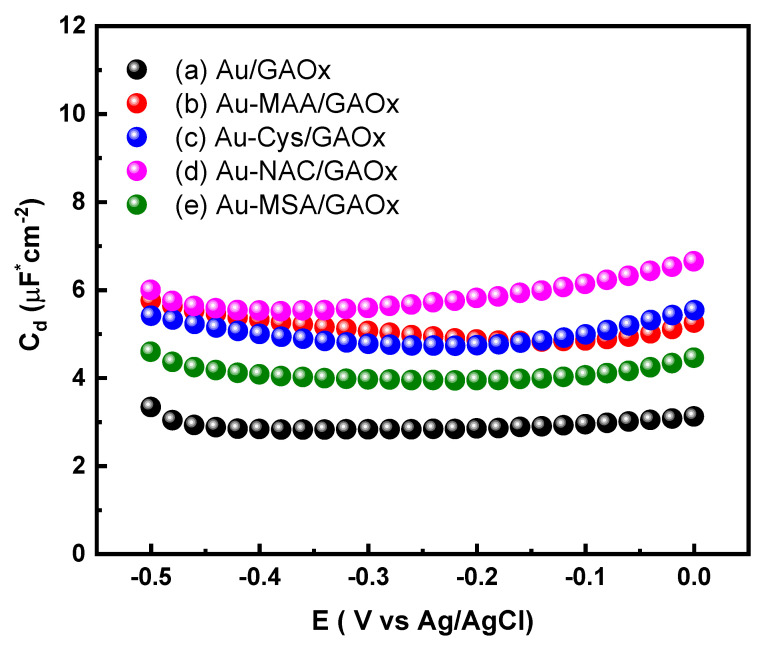
Differential capacitance curves for (a) Au/GAOx, (b) Au-MAA/GAOx, (c) Au-Cys/GAOx, (d) Au-NAC/GAOx, and (e) Au-MSA/GAOx electrodes. Measurements were performed at a frequency of 50 Hz and an amplitude of 10 mV, scanning the potential from −0.5 to 0 V.

After the thiol coating was applied to the gold surface, the adsorption of GAOx was investigated. Figure 5 presents the plots of C_dl_ vs. E (Ag/AgCl) for various electrodes: Au/GAOx (line a of Figure 5), Au-MAA/GAOx (lin b of Figure 5), Au-Cys/GAOx (line c of Figure 5), Au-NAC/GAOx (line d of Figure 5), and Au-MSA/GAOx (line e of Figure 5) within the double-layer charge potential range. A significant increase in C_dl_ values was noted for electrodes modified with both thiol groups and GAOx when compared to the Au/GAOx electrode. In these configurations, the double layer is typically characterized in existing literature as two parallel plate capacitors connected in series, where the overall capacitance (C_T_) arises from both the biomolecule layer (C_bm_) and dielectric layer (C_dL_), as detailed in Equation (4) [34,53,55]. The enhanced capacitance observed in thiol-modified electrodes incorporating GAOx is attributed to an increase in C_bm_, which results from a higher concentration of positive charges at the edges of the protein, aligning with its elevated isoelectric point (pI = 9.5).

(4) $$\frac{1}{C_{T}}=\frac{1}{C_{dL}}+\frac{1}{C_{bm}}$$

### 2.4. Zero Charge Potential

The minimal point of the C_dl_ vs. E (Ag/AgCl) curves was utilized to ascertain the zero-charge potential (E_zc_) [48]. For the uncoated Au electrode, the minimum C_dl_ value was recorded at −0.059 ± 0.015 V relative to Ag/AgCl. The E_zc_ values for the Au/MAA, Au/Cys, Au/NAC, and Au/MSA electrodes, in the absence of the enzyme, were found to be −100 ± 13, −220 ± 7, −240 ± 15, and −100 ± 18 mV vs. Ag/AgCl respectively. These E_zc_ values, along with the charge characteristics of thiols at pH 7.2, offer valuable insights into their arrangement on the electrode surface [56].

For the Au/MAA electrode, at pH 7.2 (pKa = 3.7), the carboxyl group of MAA carries a negative charge, leading to a negative surface charge when the potential is more negative than E_zc_. Conversely, when potential exceeds E_zc_ positively, the carboxyl group faces away from the electrode towards the solution. Similar behavior is anticipated for the Au/MSA electrode. In contrast, for both Au/Cys and Au/NAC electrodes, when potentials are more positive than their respective E_zc_ values, both -NH_3_^+^ and -NH_2_^+^ groups contribute positively to surface charge while exposing their carboxyl groups to solutions. When potentials fall below E_zc_ values, this orientation reverses. The determined E_zc_ values yield insights into how GAOx interacts with gold surface electrodes since GAOx has a positive charge at pH 7.2 (PI = 9.5) [57]. The adsorption of GAOx is suggested by a shift in E_zc_ toward more negative potential compared to corresponding electrodes without protein present. The measured E_zc_ values for electrodes coated with GAOx, such as Au/GAOx, Au-MAA/GAOx, Au-Cys/GAOx, Au-NAC/GAOx, and Au-MSA/GAOx, were recorded at −287 ± 17, −187 ± 11, −243 ± 10, −350 ± 14 and −230 ± 14 mV vs. Ag/AgCl, respectively. It should be noted that the potential applied also influences how the enzyme orients itself on the electrode surface.

### 2.5. Density Charge Plots

**Figure 6:**
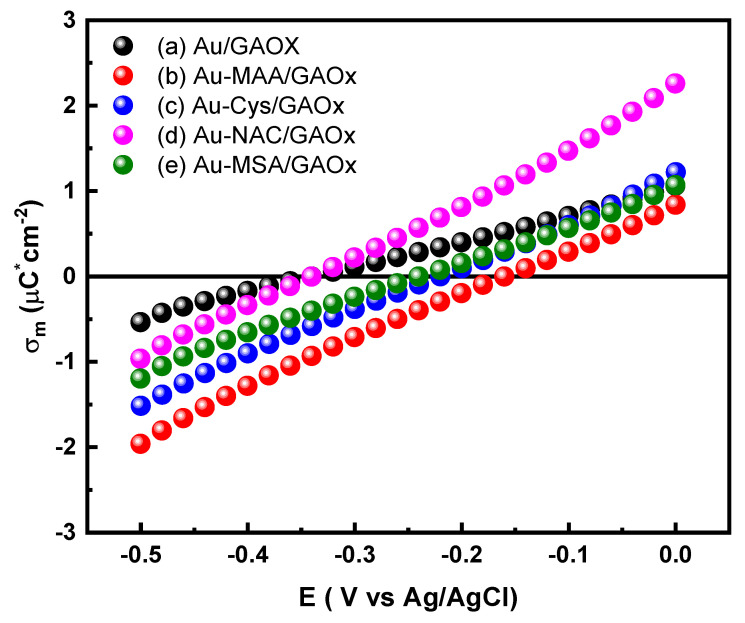
Metal charge density (σ_m_) vs. potential (E) curves for (a) Au, (b) Au-MAA/GAOx, (c) Au-Cys/GAOx (d) Au-NAC/GAOx, and (e) Au-MSA/GAOx electrodes, derived from differential capacitance measurements.

The curves representing the metal charge density (σ_m_) in relation to potential (E) for Au, Au-MAA, Au-Cys, Au-NAC, and Au-MSA electrodes in the presence of GAOx are illustrated in Figure 6. These curves were derived by integrating the differential capacitance (C_dl_) with respect to potential, as detailed by Equation (5):

(5) $$(\sigma_{m}=\int_{E_{ZC}}^{E_{0}}C_{dl}dE)$$

This analysis spans a potential range from −0.5 to 0 V vs. Ag/AgCl. For the Au/GAOx electrode shown in line a of Figure 6, σ_m_ values displayed a linear relationship with the applied potential, varying from −0.535 to 1.06 μC·cm^−2^. These measurements are notably lower than those obtained from the bare Au electrode (−3.92 to 6.24 μC·cm^−2^), suggesting that GAOx modifies the metal charge due to its positive charge at pH 7.2 (P.I = 9.5) [58]. The σ_m_ vs. E profiles for the Au-MAA/GAOx (line b of Figure 6), Au-Cys/GAOx (line c of Figure 6), and Au-MSA/GAOx (line e of Figure 6) electrodes reflect values between −1.96 to 0.84 μC·cm^−2^, −1.52 to 1.22 μC·cm^−2^, and −1.19 to 1.06 μC·cm^−2^ respectively, indicating that GAOx has a more pronounced effect on the electrode charge within these modified systems. Conversely, the Au-NAC/GAOx electrode depicted in line d of Figure 6 displays the lowest σ_m_ values among all the tested configurations; this is attributed to the bulky nature of the NAC promoter, which restricts effective interaction between the protein and electrode surface by introducing interfacial limitations. At potentials exceeding E_zc_ positively, GAOx contributes positively to the charge of the electrode, implying an orientation that brings it closer to the surface; however, at more negative potentials than E_zc_, GAOx tends to be more exposed within the solution.

### 2.6. Surface Tension Plots

**Figure 7:**
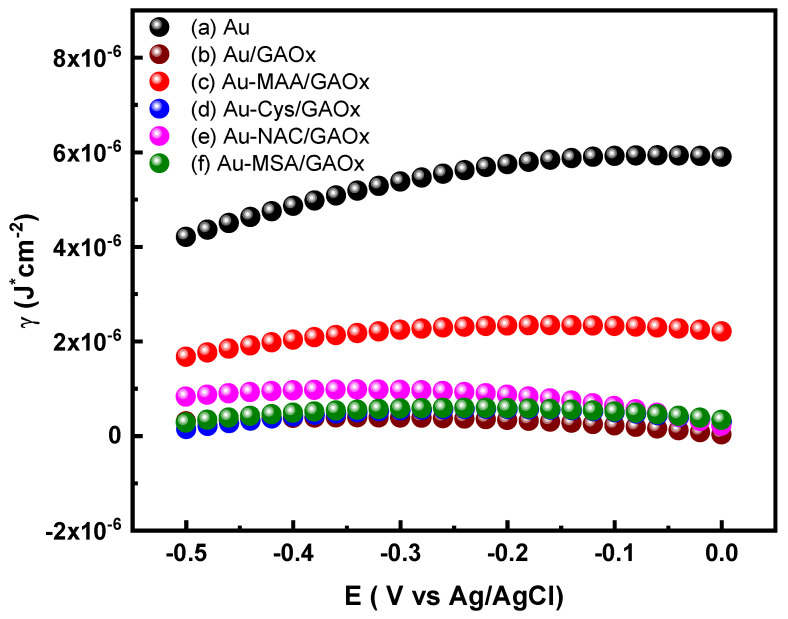
Surface tension (γ) vs. potential (E) plots for (a) Au; (b) Au/GAOx; (c) Au-MAA/GAOx; (d) Au-Cys/GAOx; (e) Au-NAC/GAOx, and (f) Au-MSA/GAOx electrodes, obtained from differential capacitance curves.

Integration of the charge density and potential curves yields the surface tension (γ) of the adsorbed film on electrode surfaces [34]. It should be noted that enzyme-modified electrodes represent intrinsically heterogeneous interfaces. Therefore, the surface tension values derived from electrocapillary analysis correspond to ensemble-averaged interfacial properties, reflecting dominant adsorption regimes rather than a perfectly homogeneous monolayer or a single, unique orientation of GAOx. Variations in surface tension indicate shifts in electrostatic and chemical interactions within the double layer [47]. Consequently, electrocapillary curves were generated for electrodes including Au, Au/GAOx, Au-MAA/GAOx, Au-Cys/GAOx, Au-NAC/GAOx, and Au-MSA/GAOx, as illustrated in Figure 7. For the Au/GAOx electrode (line b of Figure 7), a reduction in γ is noted compared to the bare Au electrode (line a of Figure 7). This phenomenon can be linked to electrostatic interactions between amino acids on the surface of the protein and ions present in solution, which results in an increased thickness of the double layer [44]. In electrodes such as Au-Cys/GAOx (line d of Figure 7), Au-NAC/GAOx (line e of Figure 7), and Au-MSA/GAOx (line f of Figure 7), the observed decrease in γ values relative to the untreated Au electrode suggests that the charged groups of GAOx enhance electrostatic interactions with ions in solution. The electrocapillary curves for both Au-Cys/GAOx (line d of Figure 7) and Au-MSA/GAOx (line c of Figure 7) display similar characteristics, suggesting comparable dominant adsorption regimes in terms of average orientation and surface population of GAOx, despite the coexistence of multiple adsorption configurations [43].

### 2.7. Surface Excess Plots

**Figure 8:**
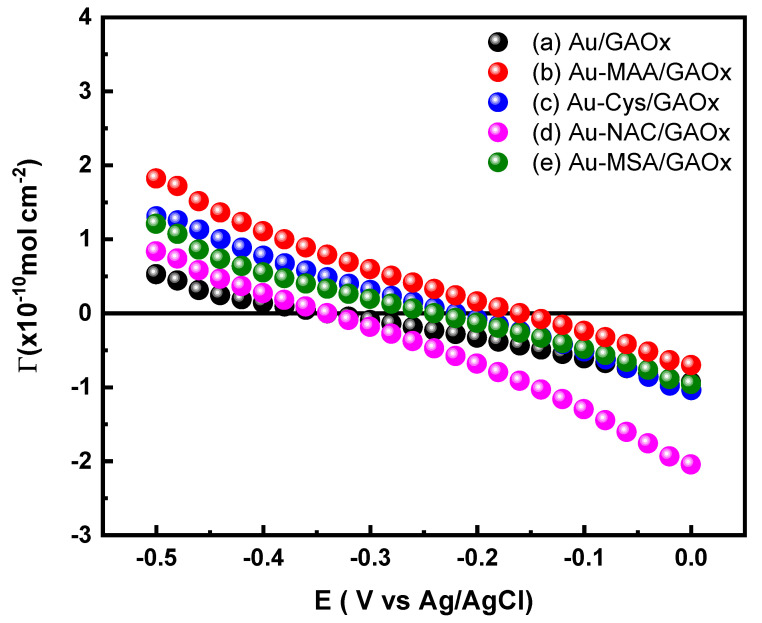
Surface excess (Γ) vs. potential (E) plots for (a) Au; (b) Au-MAA/GAOx; (c) Au-Cys/GAOx; (d) Au-NAC/GAOx; and (e) Au-MSA/GAOx electrodes, derived from differential capacitance curves.

The surface excess (Γ) of GAOx on electrodes modified with thiol was assessed through electrocapillary curves (Figure 8), employing Equation (6), where c signifies the concentration of the adsorbed species, in this instance, the enzyme.

(6) $$\Gamma =-{\left(\frac{\partial \gamma }{RT\partial \ln C}\right)}_{T,P,E}$$

For the Au/GAOx electrode (line a of Figure 8), the Γ vs. potential curves demonstrate minimal fluctuations in Γ with varying applied potentials, suggesting that the enzyme can orient itself differently without considerable changes in the amount of adsorbed enzyme as compared to its solution concentration [59]. The concentration of the absorbed enzyme appears to be lower than that in bulk solution, as indicated by the negative values of Γ. In contrast, for electrodes such as Au-MAA/GAOx (line b of Figure 8), Au-Cys/GAOx (line c of Figure 8), and Au-MSA/GAOx (line e of Figure 8), higher Γ values are observed at negative potentials when compared to the Au/GAOx electrode, implying that these promoters facilitate greater enzyme adsorption. Additionally, at a potential exceeding −0.1 V, the amounts of adsorbed GAOx across these electrodes are notably similar, indicating a uniform orientation of the enzyme. Conversely, for the Au-NAC/GAOx electrode (line d of Figure 8), Γ values are closely aligned with those of the Au/GAOx electrode at potentials lower than −0.287 V. However, at potentials higher than −0.287 V, this specific electrode exhibits the lowest Γ values compared to all other tested electrodes. This indicates that the NAC interferes with enzyme adsorption on gold surfaces by introducing interfacial limitations that hinder effective electronic coupling.

### 2.8. Gibbs Surface Excess and Adsorption Gibbs Free Energy

The Gibbs free energy of adsorption for GAOx was calculated utilizing Henry’s isotherm equations, as outlined in Equations (7) and (8) [49]. In these formulas, Π denotes the film pressure, which is influenced by the bulk concentration of the adsorbed molecules; R represents the ideal gas constant (8.314 cal·mol^−1^·K^−1^); and T standard for the temperature (298.15 K). The parameters γ_θ = 0_ and γ_θ_ signify the surface tension without adsorbed species and with adsorbed species on the electrode surface, respectively. Γ_max_ indicates the maximum surface concentration, β refers to the adsorption coefficient, and c is the bulk concentration of species in solution. Subsequently, the adsorption coefficient β was employed to determine the Gibbs free energy of adsorption using Equation (9).

(7) $$\Pi =RT\Gamma_{max}\beta c{\left(55.5\right)}^{-1}$$

(8) $$\Pi =\gamma_{\theta =0}-\gamma_{\theta }$$

(9) $$\;\;{∆G}_{add}=-RT\ln\beta$$

For the Au/GAOx electrode, the determined ∆G_add_ value was −44.9 ± 2.1 kJ·mol^−1^, suggesting that there are ion–ion interactions occurring between the protein and the electrode. Conversely, for the gold electrode that has been modified with MAA, Cys, NAC, and MSA in the presence of GAOx, the ∆G_add_ values were recorded as −39.9 ± 1.4, −41.6 ± 1.6, −42.3 ± 5.5, and −38.6 ± 1.6 kJ·mol^−1^ respectively. It should be noted that the Gibbs free energy of adsorption (∆G_add_) was derived using the Henry adsorption constant under the premise of low coverage conditions. In this scenario, the Henry isotherm provides a valuable measure of the affinity between GAOx and the surface. However, it is crucial to recognize that macromolecular adsorption may not conform to ideal behavior due to differences in surface properties and interactions that are influenced by coverage levels.

The thermodynamic parameters C_dl_, E_zc_, ϒ, and ∆G derived from these various electrodes are compiled in Table 2. These findings suggest that GAOx can adsorb onto the unmodified gold surface with less energy compared to the thiol-modified electrodes. Nevertheless, despite this easier adsorption on bare gold, electron transfer is not significantly enhanced.

It is important to note that adsorption strength and electron transfer efficiency are not necessarily correlated in a linear manner. While thermodynamic parameters such as surface excess and adsorption free energy describe the affinity and stability of GAOx at the interface, efficient electron transfer additionally requires favorable electronic coupling and an appropriate orientation of the redox-active Cu center relative to the electrode surface. Consequently, strong adsorption may stabilize configurations that are thermodynamically favorable but electronically less efficient for direct electron transfer, whereas weaker or intermediate adsorption can promote orientations that enhance electron transfer kinetics.

## 3. Materials and Methods

### 3.1. Materials

All reagents used in this study were of analytical grade. Galactose oxidase from *Dactylium dentroides* (lyophilized powder ≥ 3000 units/mg) and Thioglycolic acid (98%) were purchased from Sigma-Aldrich (St. Louis, MO, USA). Phosphate-buffered saline (PBS, pH 7.2, composed of 0.1 M sodium phosphate and 0.15 M sodium chloride) was obtained from Thermo Scientific (Waltham, Ms, USA). L-cysteine (98.5%), N-Acetyl-L-Cysteine (99%), Mercaptosuccinic acid (97%), and Mercaptoacetic acid (98%) were acquired from Sigma-Aldrich (St. Louis, MO, USA). Sulfuric acid (98%), potassium ferricyanide (99%) and potassium ferrocyanide (99.59%) were acquired from Sigma-Aldrich (St. Louis, MO, USA). Polishing materials, including 0.3 μm Micro-Cloth Polishing Cloth 8, Micro-Polish Alumina and monocrystalline diamond suspension, were obtained from Buehler (Reno County, KS, USA).

### 3.2. Electrode Preparation

After being polished with 1 μm diamond powder and 0.3 μm alumina slurry, the polycrystalline gold surface electrode (diameter = 3 mm) was cleaned with deionized water and subjected to sonication for one minute. Electrochemical cleaning was carried out in a 0.5 M H_2_SO_4_ solution by cycling the potential between −0.25 V and 1.125 V vs. Hg/HgSO_4_ at a scan rate of 0.005 V·s^−1^ for ten cycles, until the typical voltammetric profile of gold was achieved and stabilized. To verify thiol adsorption, the electrode was placed in a 5 mM solution of the relevant alkanethiol in PBS (pH 7.2), while monitoring the open circuit potential (E_oc_) over a period of fifteen minutes. The modified electrode was then thoroughly washed with deionized water to eliminate any physically adsorbed molecules. This protocol adhered to the methodology outlined in references [31]. Control experiments (Figure S6 for Cys and NAC as examples) indicate that the redox signals detected do not stem from either the gold electrode or the SAM. Additionally, a model system comprising Cu–tyrosine–histidine does not replicate the electrochemical behavior of GAOx, suggesting that the noted redox characteristics are attributable to the intact enzyme rather than isolated copper or aromatic components. This indicates that the direct transfer of electrons in GAOx is influenced not only by the presence of the Cu–tyrosine motif but also by the orientation and electronic coupling facilitated by the protein.

### 3.3. Electrochemical Measurements

Electrochemical assessments were conducted at a temperature of 25 °C utilizing a Biologic SP-300 potentiostat/galvanostat configured with a standard three-electrode cell containing 2 mL of solution. An Ag/AgCl electrode was utilized as the reference electrode, while a platinum wire served as the counter electrode. A supporting electrolyte comprising a 0.1 M phosphate buffer (pH 7.2) was employed. Before each experimental run, the solution was purged with nitrogen, and this inert atmosphere was maintained during the measurements.

The characterization of modified electrodes took place in the presence of a 5 mM Fe(CN)_6_^3−/4−^ redox probe, employing cyclic voltammetry on surfaces modified with carboxylic thiol. Voltammetric data were collected at a scan rate of 0.05 V·s^−1^, beginning from the open-circuit potential (E_oc_) and scanning in the negative direction within a potential range of −0.4 to 0.6 V against Ag/AgCl in the phosphate buffer (pH 7.2). Additionally, electrochemical impedance spectroscopy (EIS) measurements were performed at E_oc_ using an AC amplitude of 10 mV across frequencies ranging from 10 kHz to 10 mHz.

Both modified and unmodified electrodes were analyzed in an environment containing 50 µM GAOx dissolved in the supporting electrolyte of 0.1 M phosphate buffer (pH 7.2). Cyclic voltammograms were obtained across various scan rates ranging from −0.5 to 0.5 V versus Ag/AgCl [32]. Impedance measurements (ZIR) were also carried out to account for ohmic drop (iR). From these experiments, significant electrochemical parameters such as the heterogeneous electron transfer rate constant (k_s_), symmetry coefficient (α), and surface excess (Γ) were determined [45]. Differential capacitance curves were generated within a potential range of −0.5 to 0 V at a frequency of 50 Hz and an amplitude of 10 mV. All electrochemical responses were normalized concerning the electroactive surface area.

## 4. Conclusions

For the advancement in third-generation biosensors aimed at detecting galactose, it was shown that mercaptoacetic acid, L-cysteine, N-acetyl-L-cysteine, and mercaptosuccinic acid significantly enhance the electron transfer process associated with galactose oxidase (GAOx). The thermodynamic and electrochemical parameters pertinent to adsorption processes were assessed. Evidence of GAOx adsorption onto gold and thiol-modified gold electrodes was indicated by alterations in the C_dl_ curves and E_zc_ values. GAOx plays a crucial role in influencing the metal charge density at the interfaces of Au/GAOx, Au-MAA/GAOx, Au-Cys/GAOx, Au-NAC/GAOx, and Au-MSA/GAOx. Furthermore, changes in surface tension across the tested electrodes indicate that both the quantity and orientation of GAOx have a significant impact on interfacial characteristics. The electrode interface behavior implies that GAOx assumes different orientations based on the applied potential. These results illustrate that cyclic voltammetry and differential capacitance serve as effective methods for investigating adsorption processes during the fabrication of third-generation biosensors. Finally, modifications to these gold electrodes represent a rapid and flexible approach for examining how metalloenzymes orient themselves on a gold electrode concerning electrochemical parameters.

## Figures and Tables

**Table 1:** Cyclic voltammetry parameters for gold and thiol-modified electrodes at different scan rates.

Electrode	E° (mV)	∆E_p_ (mV)	k_s_ (s^−1^)	α	Γ (mol·cm^−2^)
Au-MAA/GAOx	169.7 ± 20	130 ± 10	1.04 ± 0.13	0.44 ± 0.1	6.12 × 10^−11^ ± 7.7 × 10^−12^
Au-Cys/GAOx	97.6 ± 21	200 ± 20	0.93 ± 0.09	0.57 ± 0.03	1.59 × 10^−10^ ± 5.16 ×10^−11^
Au-NAC/GAOx	176.0 ± 22	138 ± 11	1.14 ± 0.15	0.48 ± 0.05	5.01 × 10^−11^ ± 7.4 ×10^−12^
Au-MSA/GAOx	117.3 ± 19	156 ± 14	1.01 ± 0.10	0.58 ± 0.07	1.54 × 10^−10^ ± 2.3 ×10^−11^

**Table 2:** Thermodynamics parameters (C_dl_, E_zc_, ϒ and ∆G) from differential capacitance spectra.

Electrode	E_ZC_ (mV)	ϒ (J·cm^−2^)	∆G (kJ·mol^−1^)
**Au**	*−59 ± 15*	*7.55 × 10^−6^ ± 2.0 × 10^−9^*	--
**Au/GAOx**	*−287 ± 17*	*4.02 × 10^−7^ ±7.1 × 10^−9^*	*−44.9 ± 2.1*
**Au-MAA/GAOx**	*−187 ± 11*	*2.48 × 10^−6^ ± 4.9 × 10^−9^*	*−39.9 ± 1.4*
**Au-Cys/GAOx**	*−243 ± 10*	*5.49 × 10^−6^ ± 3.7 × 10^−9^*	*−41.6 ± 1.6*
**Au-NAC/GAOx**	*−350 ± 14*	*9.84 × 10^−6^ ± 2.8 × 10^−9^*	*−42.3 ± 5.5*
**Au-MSA/GAOx**	*−230 ± 14*	*5.73 × 10^−6^ ± 3.4 × 10^−9^*	*−38.6 ± 1.6*

## Data Availability

The data presented in this study are available in the article.

## Supplementary Materials

The following supporting information can be downloaded at https://www.mdpi.com/article/10.3390/molecules31040694/s1. Figure S1: (a) Cyclic voltammetric response of the Au-MAA/GAOx electrode in the presence of 50 μM of GAOx at different scan rates (25 to 2500 mV·s^−1^); (b) plot of peak currents vs. scan rate derived from the cyclic voltammograms. Figure S2: (a) Cyclic voltammetric response of the Au-NAC/GAOx electrode in the presence of 50 μM GAOx at different scan rates (25 to 2500 mV·s^−1^); (b) plot of peak currents vs. scan rate derived from the cyclic voltammograms. Figure S3: (a) Cyclic voltammetric response of the Au-MSA/GAOx electrode in the presence of 50 μM GAOx at different scan rates (25 to 2500 mV·s^−1^); (b) plot of peak currents vs. scan rate derived from the cyclic voltammograms. Figure S4: (a) Cyclic voltammetric response of the Au–Cys/GAOx electrode. (b) Cyclic voltammetric response of the Au–Cys/GAOx electrode after chronoamperometric polarization at −0.6 V vs. Ag/AgCl. (c) Cyclic voltammetric response of the Au–Cys/GAOx electrode after chronoamperometric polarization at 0.6 V vs. Ag/AgCl. All experiments were performed at a scan rate of 50 mV·s^−1^ in 0.1 M phosphate buffer (pH 7.2), as the supporting electrolyte. Figure S5: Capacitance spectra of (a) Au electrode unmodified and Au electrode modified with SAMs of (b) MAA, (c) Cys, (d) NAC, and (e) MSA. Figure S6: (1a) Unmodified Au electrode profile; (1b) modified Au–Cys electrode profile; (1c) modified Au–Cys/Cu–tyrosine–histidine electrode profile; (1d) modified Au–Cys/GAOx electrode. (2a) Unmodified Au electrode profile; (2b) modified Au–NAC electrode profile; (2c) modified Au–NAC/Cu–tyrosine–histidine electrode profile; (2d) modified Au–NAC/GAOx electrode. All measurements were performed at a scan rate of 50 mV·s^−1^ in 0.1 M phosphate buffer (pH 7.2) as the supporting electrolyte.

## Abbreviations

The following abbreviations are used in this manuscript:

GAOx	Galactose oxidase
SAMs	self-assembled monolayers
NAC	N-Acetyl-L-cysteine
MSA	Mercaptosuccinic acid
MAA	Mercaptoacetic acid
Cys	L-cysteine
