# Supplementary material for: Thermodynamic and Kinetic Analysis of Galactose Oxidase Direct Electron Transfer on Carboxyl-Terminated SAM-Modified Gold Electrodes
Source: Molecules. 2026 Feb 17;31(4):694. doi: 10.3390/molecules31040694 (PMC12943723; doi:10.3390/molecules31040694)
Supplement: Supplementary file 1 [file molecules-31-00694-s001.zip › molecules-4134515-supplementary.pdf]

Supplementary Materials

Thermodynamic and Kinetic Analysis of Galactose Oxidase Direct Electron Transfer on Carboxyl-Terminated SAM-Modified Gold Electrodes

Martha Leticia Jiménez-González [1,2], Gilberto Rocha-Ortiz [1], Luis Gabriel Talavera-Contreras [1], Jose de Jésus Gómez-Guzmán [3], René Antaño-Lopez [1], Marisela Cruz-Ramírez [4,*] and Luis Ortiz-Frade [1,*]

[1] Departamento de Electroquímica, Centro de Investigación y Desarrollo Tecnológico en Electroquímica S.C. Parque Tecnológico Querétaro, Sanfandila, Pedro de Escobedo 76703, Querétaro, Mexico
[2] Departamento de Química, Universidad Autónoma de Aguascalientes, Av. Universidad 940, Aguascalientes 20100, Ags, Mexico
[3] Departamento Académico de Biotecnológicas y Ambientales, Universidad Autónoma de Guadalajara. Av. Patria 1201, Zapopan 45129, Mexico
[4] Colegio de Bachilleres, Universidad Autónoma de Querétaro, Campus San Juan del Río, Calle Corregidora No. 4, Colonia Centro, San Juan del Río 76800, Querétaro, Mexico
* Correspondence: marisela.cruz@uaq.mx (M.C.-R.); lortiz@cideteq.mx or laofrade@gmail.com (L.O.-F.); Tel.: +52-442-211-6065

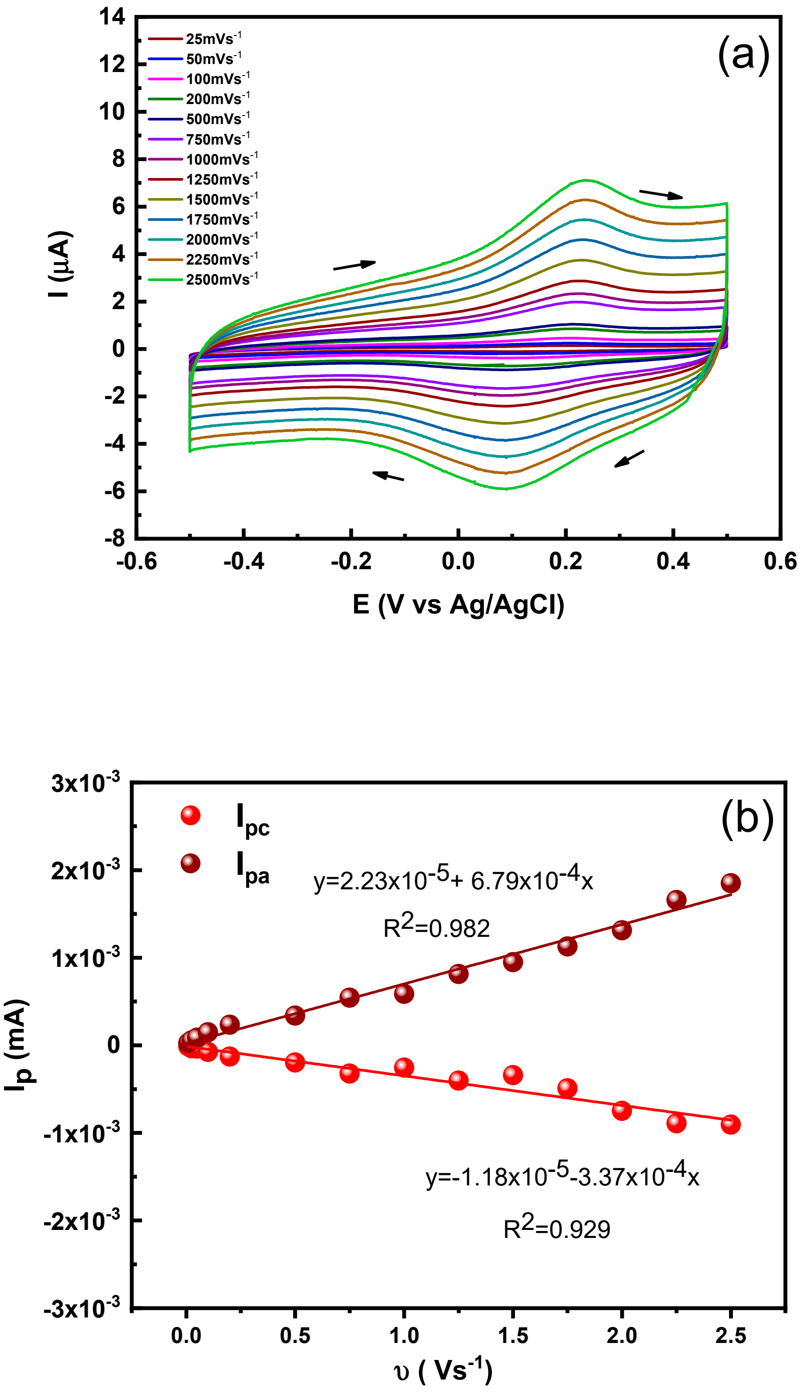

Figure S1. (a) Cyclic voltammetric response of the Au-MAA/GAOx electrode in the presence of 50 μM of GAOx at different scan rates (25 to 2500 mV·s-1); (b) plot of peak currents vs. scan rate derived from the cyclic voltammograms.

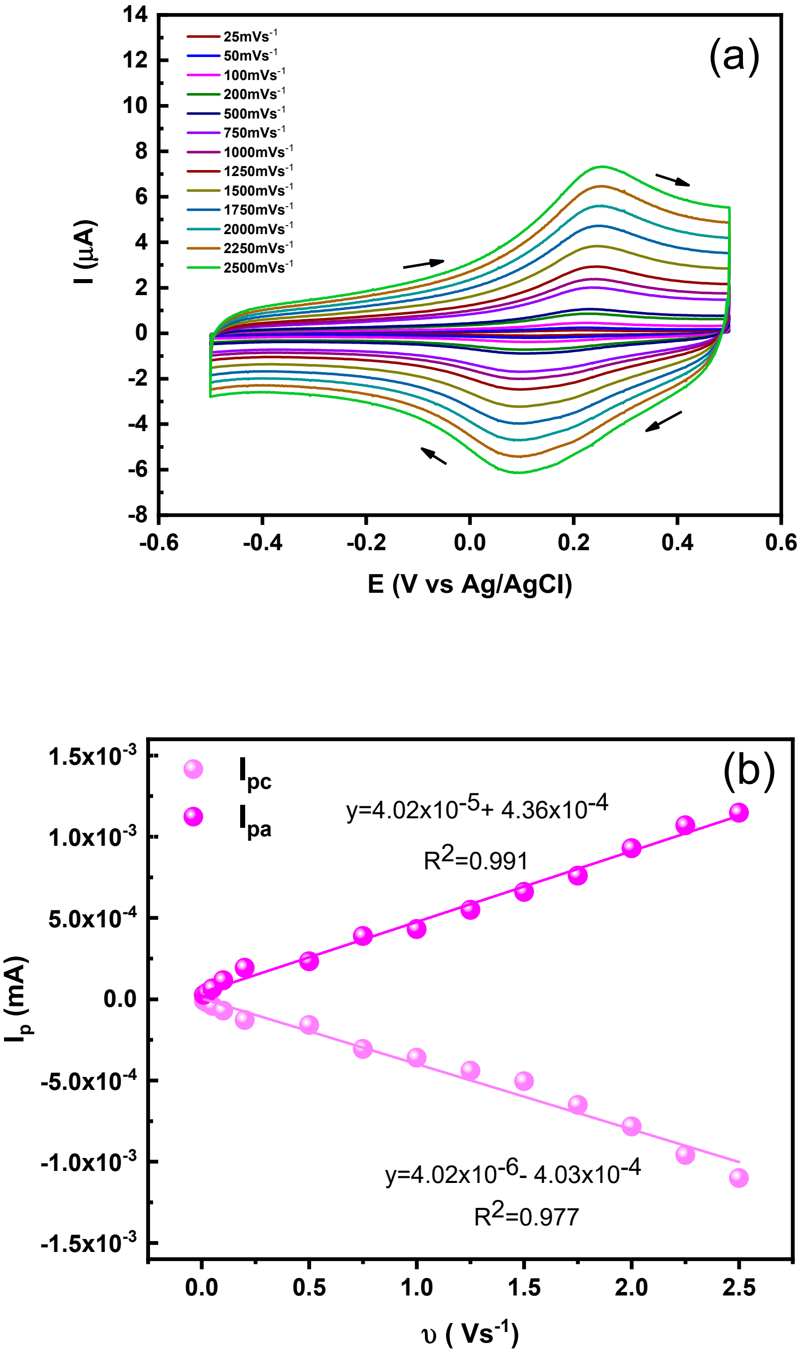

Figure S2. (a) Cyclic voltammetric response of the Au-NAC/GAOx electrode in the presence of 50 μM GAOx at different scan rates (25 to 2500 mV·s-1); (b) plot of peak currents vs. scan rate derived from the cyclic voltammograms.

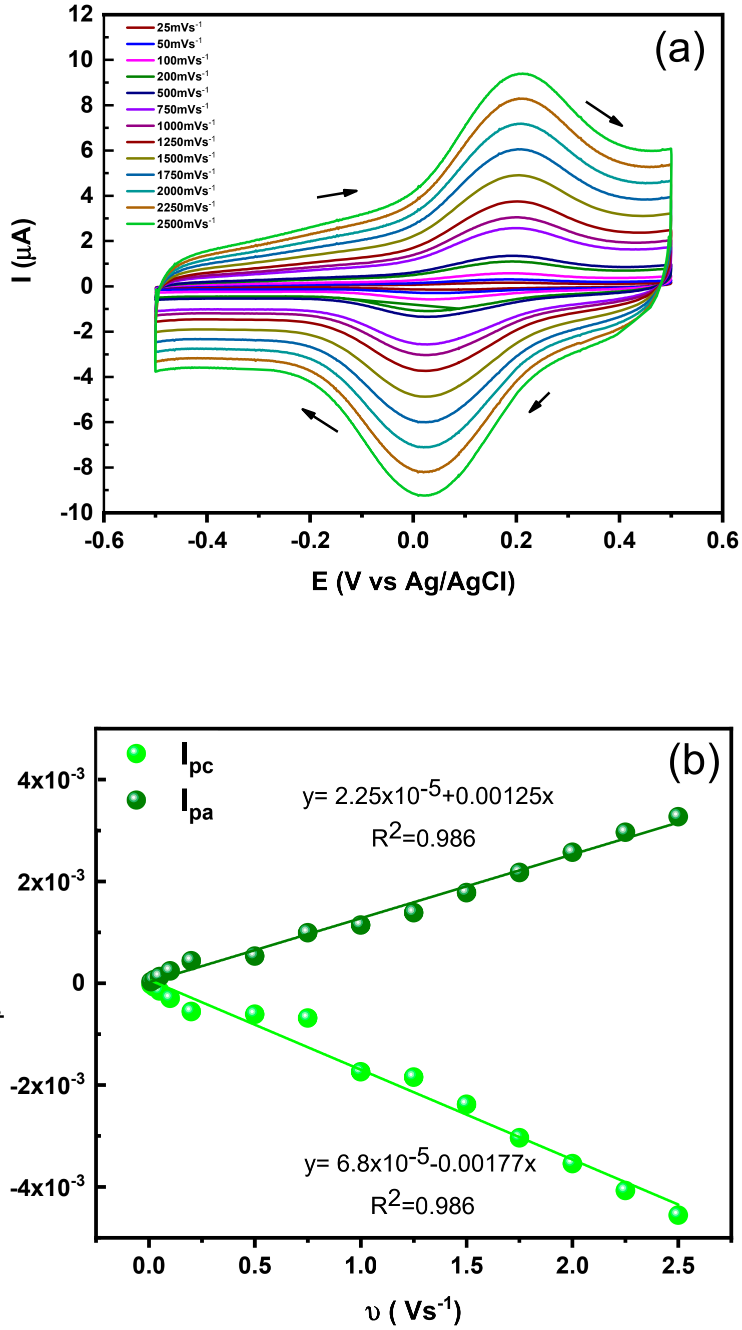

Figure S3. (a) Cyclic voltammetric response of the Au-MSA/GAOx electrode in the presence of 50 μM GAOx at different scan rates (25 to 2500 mV·s-1); (b) plot of peak currents vs. scan rate derived from the cyclic voltammograms.

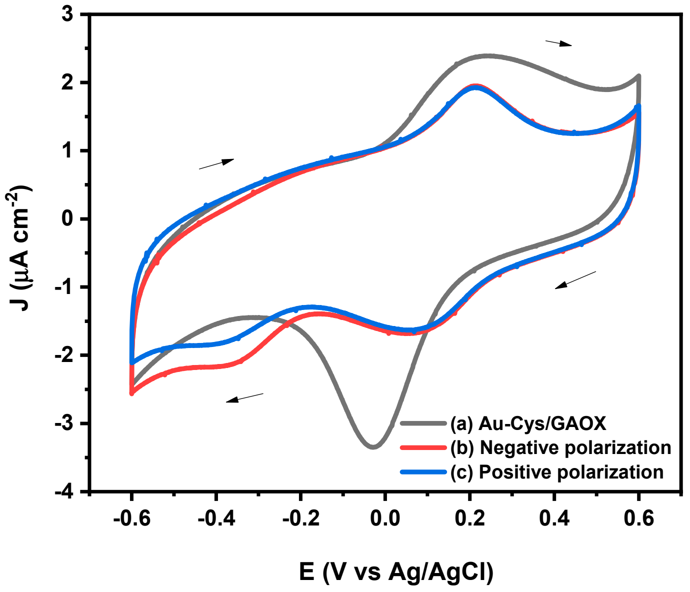

Figure S4. (a) Cyclic voltammetric response of the Au–Cys/GAOx electrode. (b) Cyclic voltammetric response of the Au–Cys/GAOx electrode after chronoamperometric polarization at −0.6 V vs Ag/AgCl. (c) Cyclic voltammetric response of the Au–Cys/GAOx electrode after chronoamperometric polarization at 0.6 V vs Ag/AgCl. All experiments were performed at a scan rate of 50 mVs^{-1} in 0.1M phosphate buffer (pH 7.2), as the supporting electrolyte.

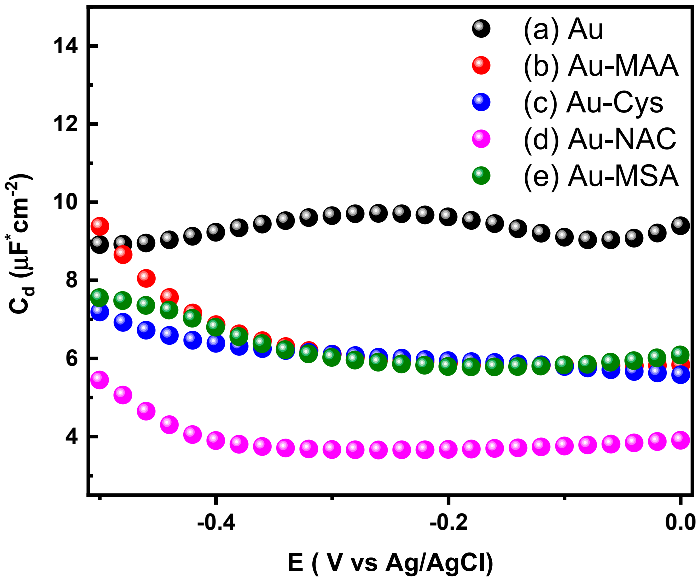

Figure S5. Capacitance spectra of (a) Au electrode unmodified and Au electrode modified with SAMs of (b) MAA, (c) Cys, (d) NAC, and (e) MSA.

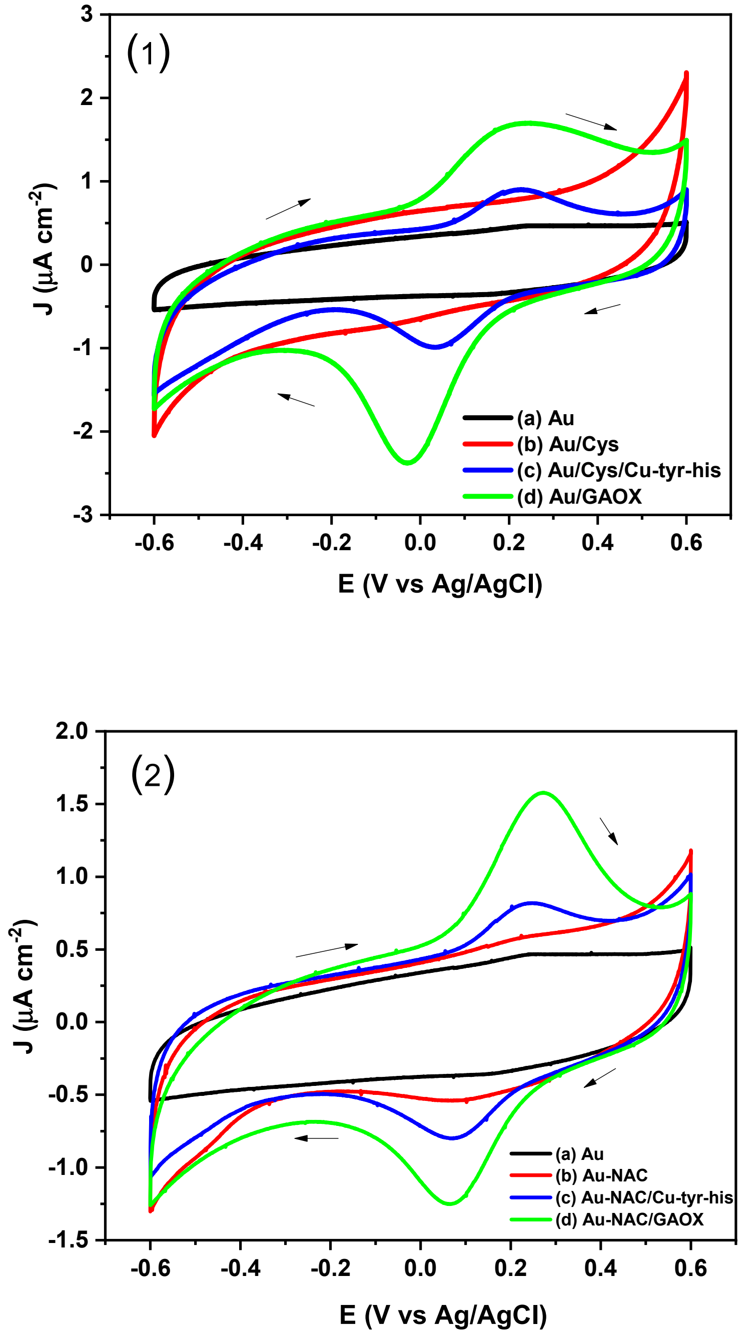

Figure S6. (1a) Unmodified Au electrode profile; (1b) modified Au–Cys electrode profile; (1c) modified Au–Cys/Cu–tyrosine–histidine electrode profile; (1d) modified Au–Cys/GAOx electrode. (2a) Unmodified Au electrode profile; (2b) modified Au–NAC electrode profile; (2c) modified Au–NAC/Cu–tyrosine–histidine electrode profile; (2d) modified Au–NAC/GAOx electrode. All measurements were performed at a scan rate of 50 mV s^{-1} in 0.1 M phosphate buffer (pH 7.2) as the supporting electrolyte.
